# Supplementary material for: Enhanced peripheral nerve regeneration by mechano-electrical stimulation
Source: NPJ Regen Med. 2023 Oct 17;8:57. doi: 10.1038/s41536-023-00334-y (PMC10582163; doi:10.1038/s41536-023-00334-y)
Supplement: Supplementary file 1 — Supplementary Information [file 41536_2023_334_MOESM1_ESM.pdf]

Supplementary Information

Enhanced Peripheral Nerve Regeneration by Mechano-electrical Stimulation.

Youyi Tai[1], Thamidul Islam Tonmoy[1], Shwe Win[1], Natasha T. Brinkley[1], B. Hyle Park[1], Jin Nam[1]*

[1]Department of Bioengineering, University of California, Riverside, CA 92521

*Corresponding author: Jin Nam, Ph.D., jnam@engr.ucr.edu

Keywords: peripheral nerve regeneration, mechano-electrical stimulation, piezoelectric, electrospun P(VDF-TrFE)

Supplementary Table 1. Primer sets for rt-qPCR analysis of PC12 and RSC96 cells.

Marker	Primer	Forward	Reverse
Housekeeping	*Rps18*	5’-CCCGAGAAGTTTCAGCACATC-3’	5’-ATGGCAGTGATAGCGAAGGCT-3’
	Gapdh	5’-GCAAGAGAGAGGCCCTCAG-3’	5’-TGTGAGGGAGATGCTCAGTG-3’
Neuronal markers	*Tubb3*	5'-GGGCCAAGTTCTGGGAAGTC-3'	5'-AGTCGCCCACGTAGTTGCC-3'
	Map2	5'-AAGCCATTGTGTCCGAACCA-3'	5'-GAGCGGAAGAGCAGTTTGTCA-3'
	Chat	5'-AGCCTTCCTAAGCCTCTACTG-3'	5'-CTAAGCACACCAGAGATGAGG-3'
Schwann cell markers	*Ngf*	5’-TTCCAGGCCCATGGTACAAT-3’	5’-AAACTCCCCCATGTGGAAGAC-3’
	Krox20	5’-TGCGCCTAGAAACCAGACCTT-3’	5’-ATGCCCGCACTCACAATATTG-3’
	Ncam-1	5’-TGGAACGCCGAGTACGAAGTA-3’	5’-TGAACACGAAGTGAGCTGCCT-3’
	Pmp22	5’-TGTACCACATCCGCCTTGG-3’	5’-GAGCTGGCAGAAGAACAGGAAC-3’

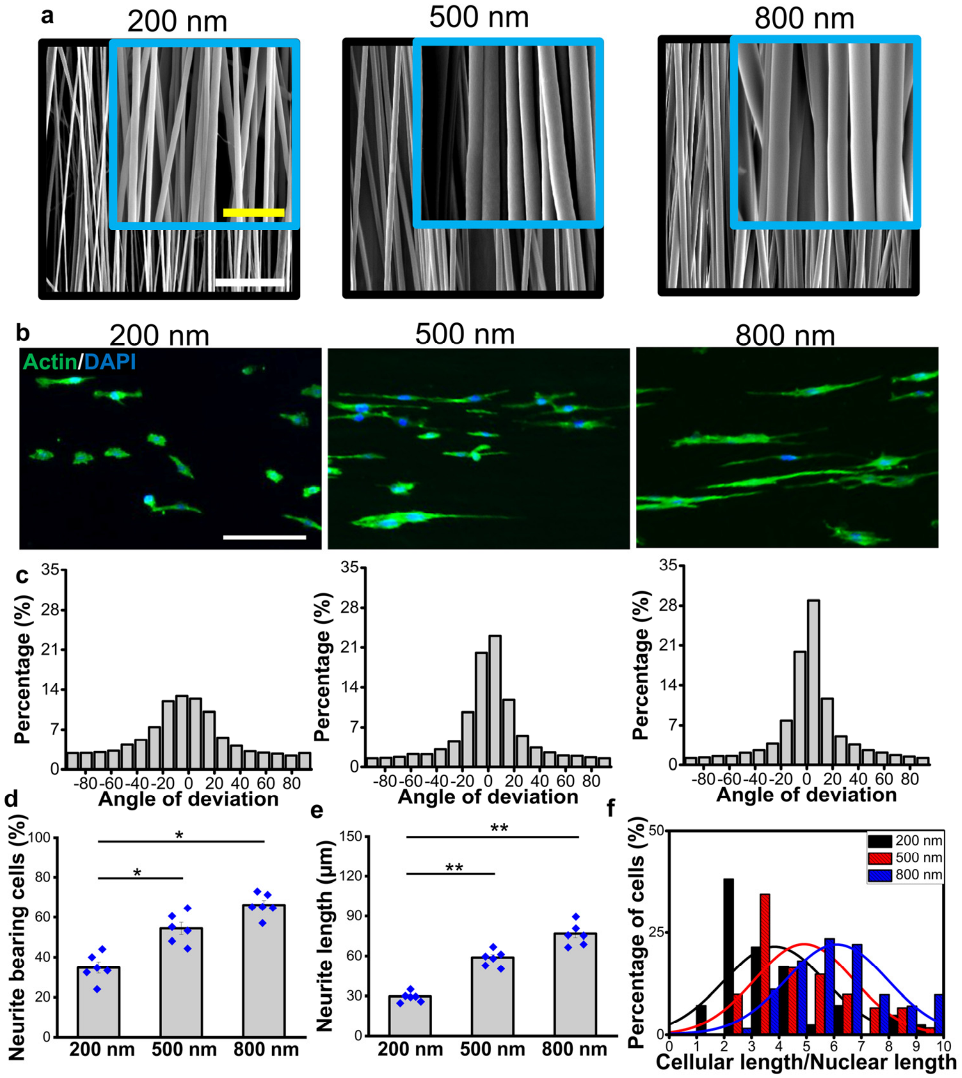

Supplementary Figure 1. Effects of different fiber diameters on neurite formation and extension. **a** Scanning electrospun microscopic (SEM) images of electrospun P(VDF-TrFE) scaffolds with an aligned fibrous structure having 200 nm, 500 nm, or 800 nm average fiber diameter (Yellow scale bar: 2 µm, White scale bar: 6 µm). **b** Immunofluorescence images depicting cellular morphology of PC12 cells, which were cultured on electrospun aligned P(VDF-TrFE) nanofibers with average diameters of 200 nm, 500 nm, and 800 nm (scale bar: 100 µm). **c** Directionality histograms of the cells on the scaffolds having various average fiber diameters of (left) 200, (middle) 500, and (right) 800 nm. **d** The percentage of cell population bearing neurites, **e** average neurite length, and **f** the percentage of neurite-bearing cells possessing a certain range of cellular length-to-nuclei length ratio, quantified from immunofluorescence images in **b**. Error bars represent standard error of means (n=6). * and ** denote statistical significance of $p < 0.05$ and $p < 0.01$, respectively. Six images were used for quantification in each graph.

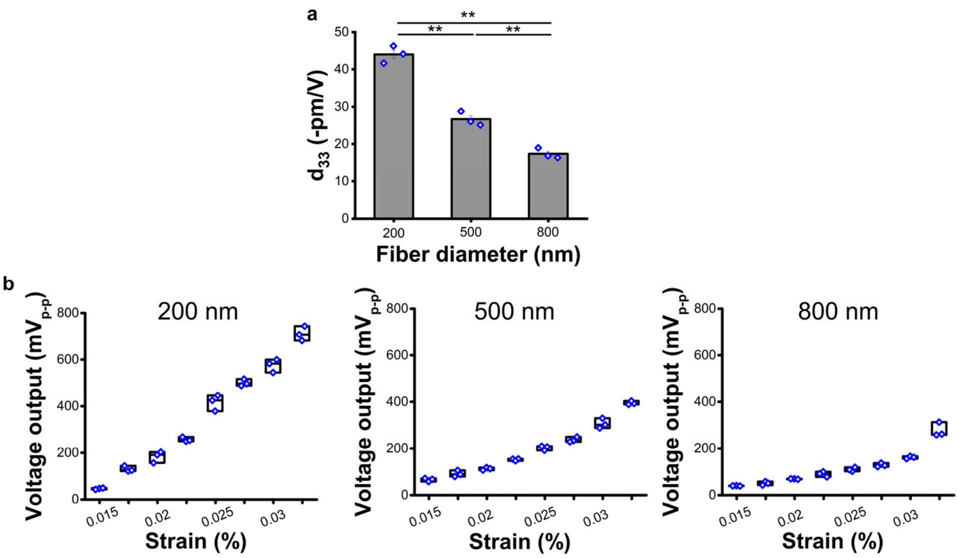

Supplementary Figure 2. Morphological and piezoelectrical characterization of electrospun P(VDF-TrFE) having various fiber diameters. a Piezo-force microscopy (PFM) measurements of individual electrospun P(VDF-TrFE) fibers having approximately 200, 500, or 800 nm fiber diameters. **b** The electric response of the electrospun P(VDF-TrFE) scaffolds with an average fiber diameter of 200, 500, or 800 nm (from left to right) under hydro-acoustic actuation that induces longitudinal strains from 0.015% to 0.0325%. Error bars represent standard error of means (n=3). ** denotes statistical significance of $p < 0.01$. The d_{33} value and the voltage outputs of 3 individual fibers and 3 scaffolds at each fiber diameter were measured, respectively.

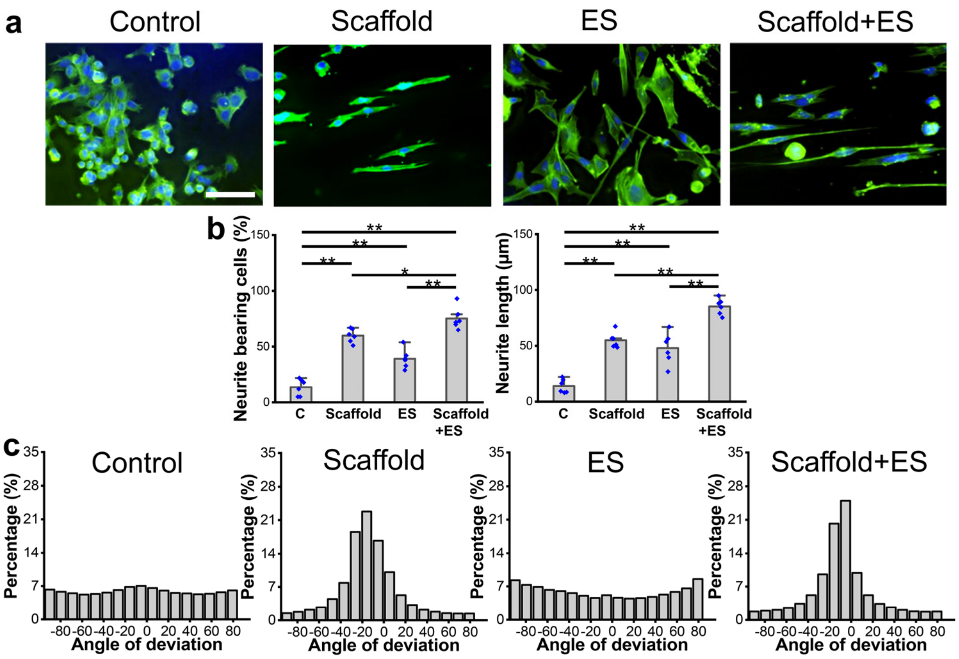

Supplementary Figure 3. Effects of aligned P(VDF-TrFE) scaffold morphology, electrical stimulation, and the combination of both on neuronal differentiation of PC12 cells. Cells were cultured for a total duration of 5 days. The aligned P(VDF-TrFE) scaffold having 500 nm fiber diameter was used and a magnitude of 200 mV_{p-p} electrical stimulation (ES, once per day for 2 hours from day 3 to day 5) was applied. Cells cultured on typical tissue culture plates with electrodes were used as a control. **a** Immunofluorescence images showing cellular morphology of PC12 cells, which were cultured on tissue culture well without ES (Control), aligned P(VDF-TrFE) scaffold without ES (Scaffold), tissue culture well with ES (ES), and aligned P(VDF-TrFE) scaffold with ES (Scaffold+ES) (scale bar: 50 μm). **b** The percentage of cell population bearing neurites and the average neurite length quantified from immunofluorescence images in **a**. **c** Directionality histograms of the cells under the Control, Scaffold, ES, and Scaffold+ES conditions. Error bars represent standard error of means (n=6). * and ** denote statistical significance of $p < 0.05$ and $p < 0.01$, respectively. Six images were used for quantification in each graph.

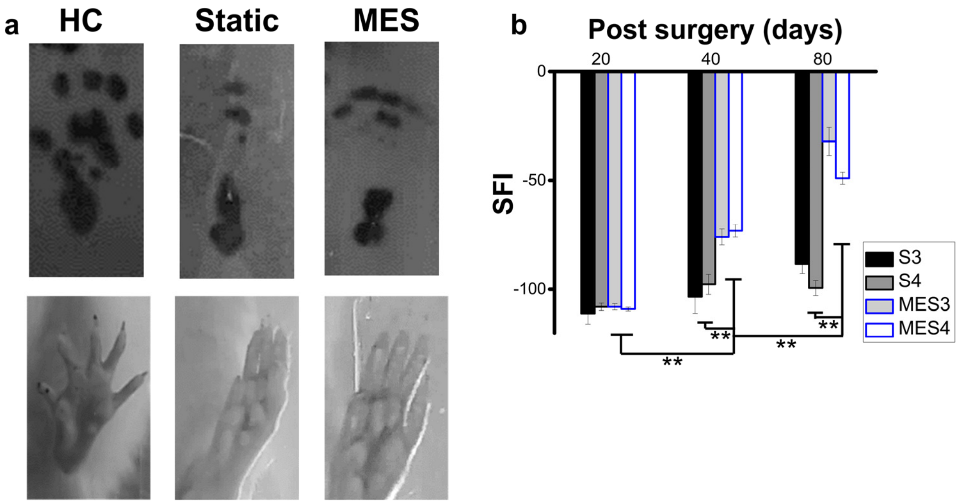

Supplementary Figure 4. Walking track analysis. Representative footprint images of walking track analysis and the corresponding sciatic functional index (SFI) quantification. a Rat footprints (top) collected for SFI measurements and photographs of rat paws (bottom) while the rats of healthy control (HC), static (Static) and MES conditions were walking through a transparent track. **b** Representative SFI data showing the functional motion recovery after the surgery on day 20, day 40, and day 80. Error bars represent standard error of means (n=3). ** denotes statistical significance of $p < 0.01$. 3 walk tracks for each rat were used for quantification.

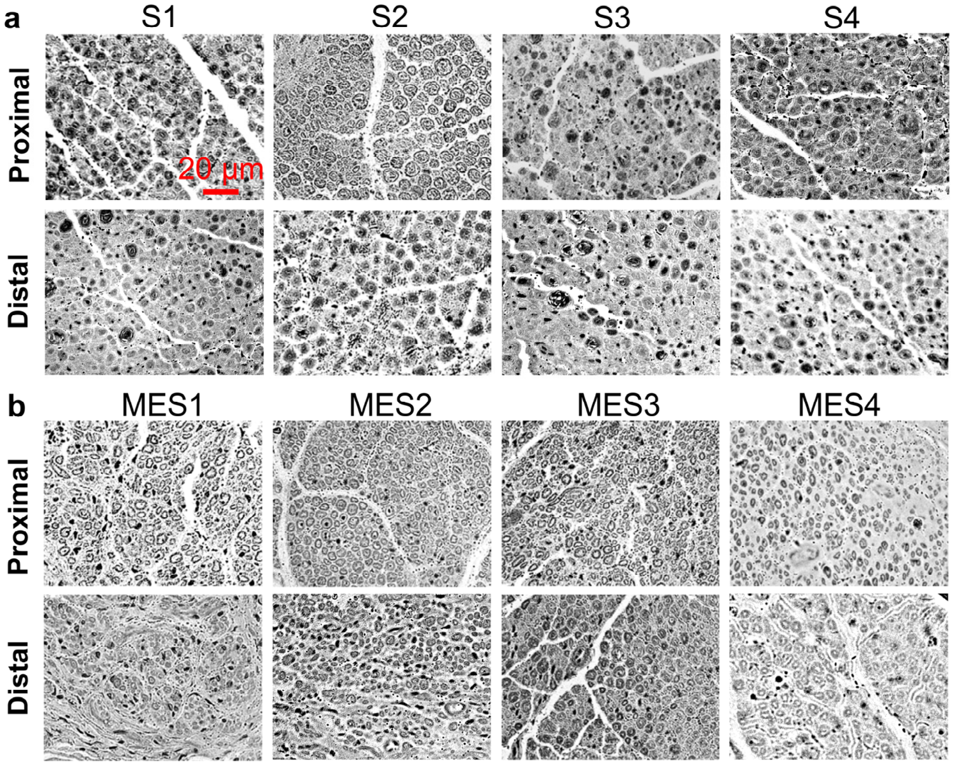

Supplementary Figure 5. Cross-sectional images of sciatic nerves (proximal and distal) in all experimental rats. Cross-sectional images of proximal and distal ends of the 4 rats in **a** Static condition and **b** MES condition.

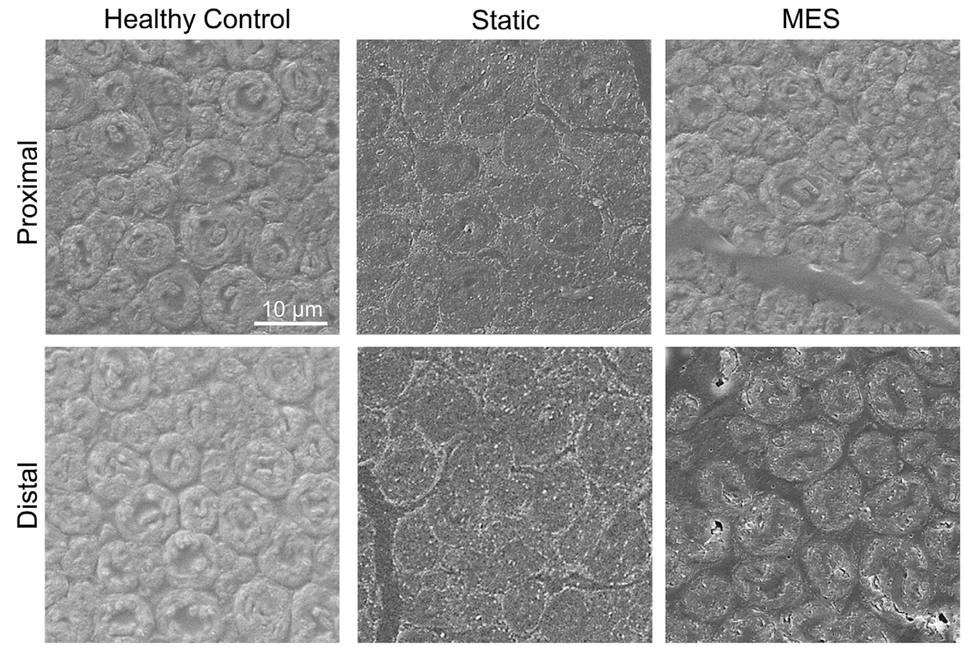

Supplementary Figure 6. Representative scanning electron microscopy (SEM) images of rat sciatic nerve.

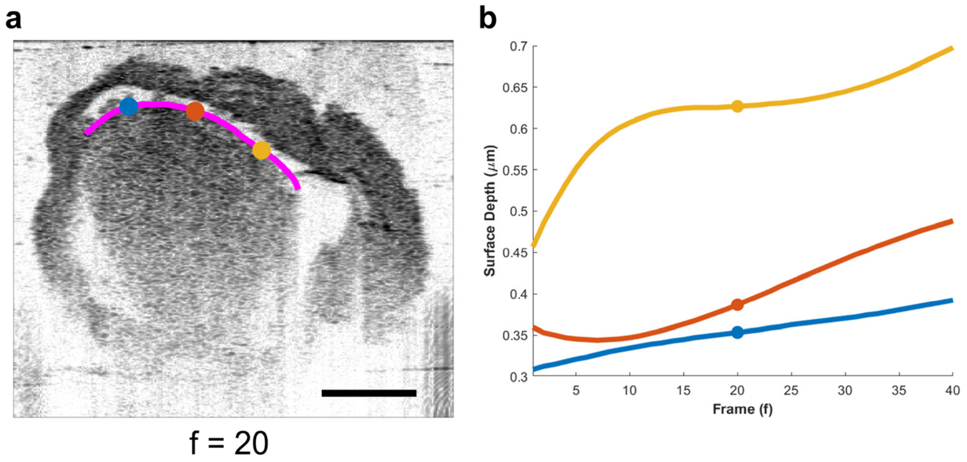

Supplementary Figure 7. Procedure for determining the nerve surface. a The manually traced nerve surface (magenta line) is shown on a cross-sectional intensity image. In the intensity images, black and white color represent high and low back reflected light intensity, respectively. For ease of presentation, the depths at three distinct A-lines are plotted with blue, orange, and yellow dots. **b** The low-order polynomial fit of the depth values at different A-lines to obtain the nerve surface in every frame. Scale bar: 0.5 mm.

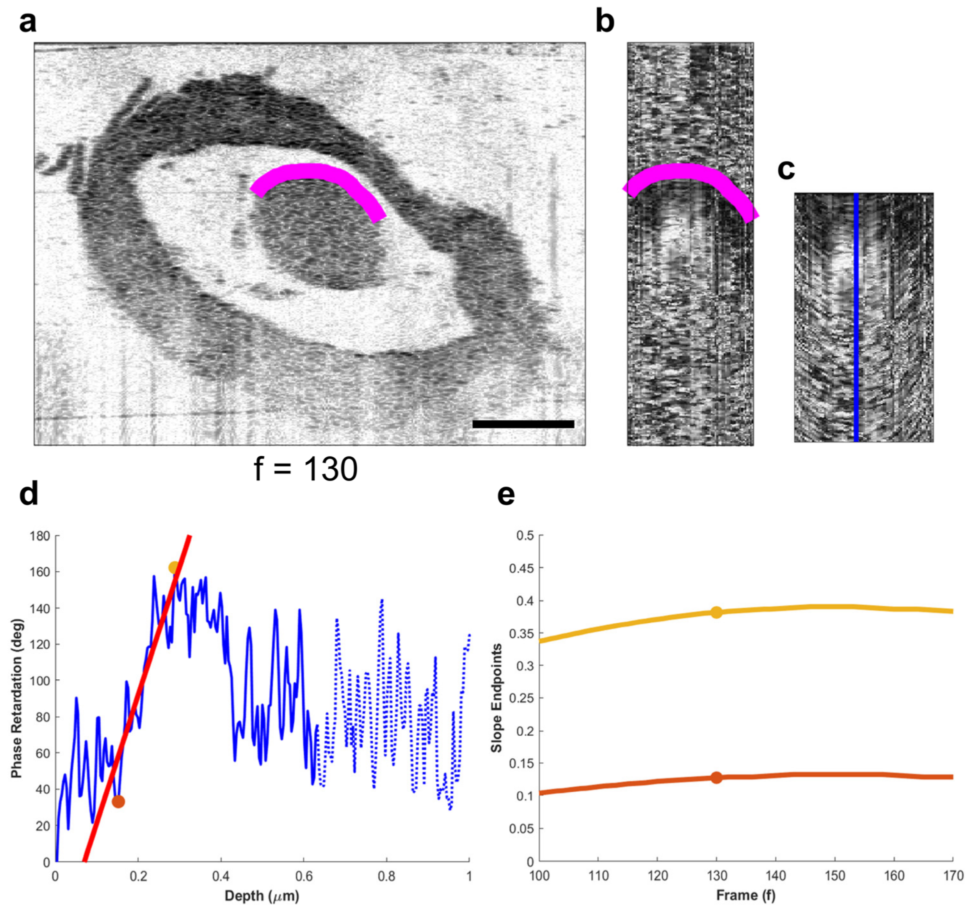

Supplementary Figure 8. Procedure for determining the endpoints for measuring the slope of the phase retardation curve. a, **b** Cross-sectional intensity image and the corresponding cumulative phase retardation image of the nerve region. In the intensity image, black and white color represent high and low back reflected light intensity, respectively. In the phase retardation image, black and white represent 0 and 180 deg phase retardation, respectively. The magenta lines in **a** and **b** represent the surface of the nerve. **c** The height-adjusted cumulative phase retardation image obtained by flattening the nerve surface in **b** so that the depth of the surface remains at 0. **d** The phase retardation vs depth curve taken from the A-line highlighted by the vertical blue line in **c**. The orange and yellow dots in **d** denote the manually identified endpoints of the rising portion of the phase retardation curves. The broken lines in **d** highlight the regions where the degree of polarization uniformity is low (< 0.5). The red line in **d** is the linear least-square fit line from which the slope of the curve is obtained. **e** The endpoints across every ten frames in a volume section fit with a low-order polynomial to obtain the endpoints in every frame. Scale bar: 0.5 mm.

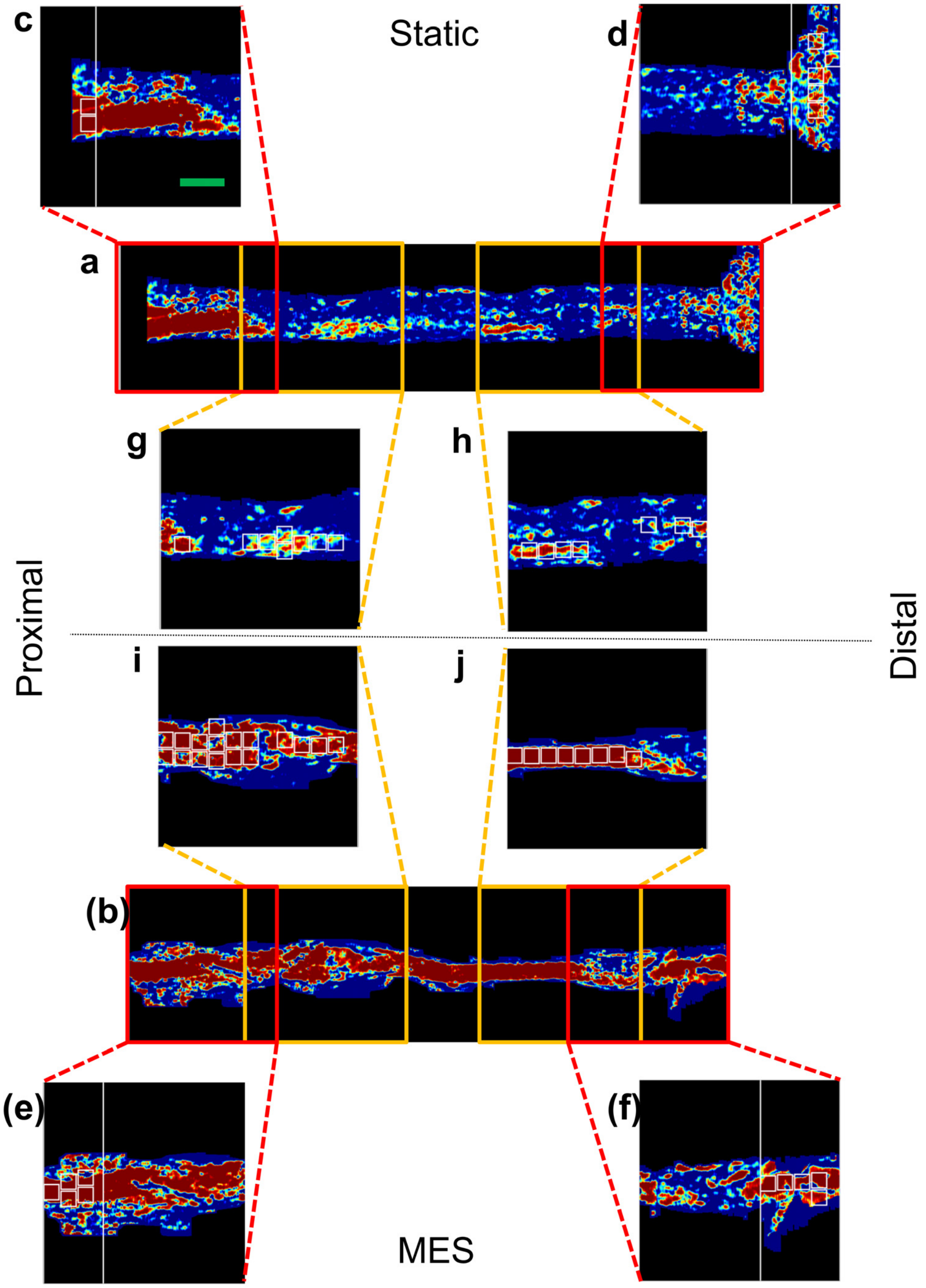

Supplementary Figure 9. Example images demonstrating the tiles for quantitative analysis. a and **b** are the enface phase retardation slope images of the whole length of the samples S2 and MES2. In these images, blue to red color indicates low to high phase retardation in the range [0.07 0.2] deg μm^{-1}. The red rectangles in **a** and **b** highlight the volume sections used for quantitative comparison with the histological measurement. The yellow rectangles in **a** and **b** highlight the volume sections used for the quantitative comparison between the Static and MES groups. **c-j** shows the zoomed-in images of the corresponding highlighted sections in **a** and **b**. The vertical white lines in **c-f** denote the beginning/end of the conduit. The tiles in white rectangles in **c-j** highlight regions from where the average slopes were measured. The mean and standard error of these measurements across the tiles were calculated for both the proximal and distal ends of the different rats and were used for the quantitative comparison. Scale bar: 1 mm.

Supplementary Video 1: **Representative rat walking video under the MES condition on day 80 post-surgery.** The toe-spreading of the injury site was highlighted by the red rectangle.

Supplementary Video 2: **Representative rat walking video under the static condition on day 80 post-surgery.** The experimental rat, under the Static condition, walked through the track with less toe-spreading (highlighted by red rectangle), as compared to that under the MES condition, indicating insufficient functional nerve regeneration when there was no mechano-electrical stimulation.
