# Supplementary material for: Enhanced peripheral nerve regeneration by mechano-electrical stimulation
Source: NPJ Regen Med. 2023 Oct 17;8:57. doi: 10.1038/s41536-023-00334-y (PMC10582163; doi:10.1038/s41536-023-00334-y)
Supplement: Supplementary file 4 — Reporting summary [file 41536_2023_334_MOESM4_ESM.pdf]

nature portfolio

Corresponding author(s): Jin Nam

Last updated by author(s): Sep 17, 2023

Reporting Summary

Nature Portfolio wishes to improve the reproducibility of the work that we publish. This form provides structure for consistency and transparency in reporting. For further information on Nature Portfolio policies, see our Editorial Policies and the Editorial Policy Checklist.

Statistics

For all statistical analyses, confirm that the following items are present in the figure legend, table legend, main text, or Methods section.

n/a	Confirmed	
☐	☒	The exact sample size (*n*) for each experimental group/condition, given as a discrete number and unit of measurement
☐	☒	A statement on whether measurements were taken from distinct samples or whether the same sample was measured repeatedly
☐	☒	The statistical test(s) used AND whether they are one- or two-sided<br>*Only common tests should be described solely by name; describe more complex techniques in the Methods section.*
☒	☐	A description of all covariates tested
☐	☒	A description of any assumptions or corrections, such as tests of normality and adjustment for multiple comparisons
☐	☒	A full description of the statistical parameters including central tendency (e.g. means) or other basic estimates (e.g. regression coefficient) AND variation (e.g. standard deviation) or associated estimates of uncertainty (e.g. confidence intervals)
☐	☒	For null hypothesis testing, the test statistic (e.g. *F*, *t*, *r*) with confidence intervals, effect sizes, degrees of freedom and *P* value noted<br>*Give P values as exact values whenever suitable.*
☒	☐	For Bayesian analysis, information on the choice of priors and Markov chain Monte Carlo settings
☒	☐	For hierarchical and complex designs, identification of the appropriate level for tests and full reporting of outcomes
☐	☒	Estimates of effect sizes (e.g. Cohen's *d*, Pearson's *r*), indicating how they were calculated

Our web collection on statistics for biologists contains articles on many of the points above.

Software and code

Policy information about availability of computer code

Data collection	the Matlab software was used to collect data from Optical Coherence Tomography (OCT). Correlating code was available in Github.
Data analysis	the Matlab software was used to analyze data from Optical Coherence Tomography (OCT). Correlating code was available in Github.

For manuscripts utilizing custom algorithms or software that are central to the research but not yet described in published literature, software must be made available to editors and reviewers. We strongly encourage code deposition in a community repository (e.g. GitHub). See the Nature Portfolio guidelines for submitting code & software for further information.

Data

Policy information about availability of data

All manuscripts must include a data availability statement. This statement should provide the following information, where applicable:

- Accession codes, unique identifiers, or web links for publicly available datasets
- A description of any restrictions on data availability
- For clinical datasets or third party data, please ensure that the statement adheres to our policy

The datasets used and/or analyzed during the current study available from the corresponding author on reasonable request.

Research involving human participants, their data, or biological material

Policy information about studies with human participants or human data. See also policy information about sex, gender (identity/presentation), and sexual orientation and race, ethnicity and racism.

Reporting on sex and gender	None
Reporting on race, ethnicity, or other socially relevant groupings	None
Population characteristics	None
Recruitment	None
Ethics oversight	None

Note that full information on the approval of the study protocol must also be provided in the manuscript.

Field-specific reporting

Please select the one below that is the best fit for your research. If you are not sure, read the appropriate sections before making your selection.

☒ Life sciences ☐ Behavioural & social sciences ☐ Ecological, evolutionary & environmental sciences

For a reference copy of the document with all sections, see nature.com/documents/nr-reporting-summary-flat.pdf

Life sciences study design

All studies must disclose on these points even when the disclosure is negative.

Sample size	The sample sizes were selected based on prior experience and practical consideration. These include 1. previous similar studies that used comparable sample sizes. 2. Expected effect size based on previous studies where a substantial effect could be detected by a smaller sample size.
Data exclusions	No data was excluded
Replication	1. Documentation of detailed standard operating procedure for each experiment involved in the study.<br>2. Repeated experimenting and analysis based on the documented protocols for the verification of the reproducibility.<br>3. Statistical analysis<br>All experimental results could be reproduced.
Randomization	All samples, organisms were allocated randomly into each experimental group.
Blinding	Investigators were blinded when running data and imaging analysis.

Reporting for specific materials, systems and methods

We require information from authors about some types of materials, experimental systems and methods used in many studies. Here, indicate whether each material, system or method listed is relevant to your study. If you are not sure if a list item applies to your research, read the appropriate section before selecting a response.

Materials & experimental systems

n/a	Involved in the study	
☐	☒	Antibodies
☐	☒	Eukaryotic cell lines
☒	☐	Palaeontology and archaeology
☐	☒	Animals and other organisms
☒	☐	Clinical data
☒	☐	Dual use research of concern
☒	☐	Plants

Methods

n/a	Involved in the study	
☒	☐	ChIP-seq
☒	☐	Flow cytometry
☒	☐	MRI-based neuroimaging

Antibodies

Antibodies used	anti-NGF Santa Cruz sc-32300 monoclonal anti-NF200 DSHB monoclonal anti-MBP Bio-rad monoclonal
Validation	Company validated species reactive to rat and mouse. Antibody also works in the manuscript

Eukaryotic cell lines

Policy information about cell lines and Sex and Gender in Research

Cell line source(s)	PC12 (mouse) and RSC96 (rat)
Authentication	Cell lines were not authenticated in the current lab but they were directly ordered from the ATCC company where they were thoroughly authenticated.
Mycoplasma contamination	All cell lines tested negative for mycoplasma contamination.
Commonly misidentified lines (See ICLAC register)	None

Animals and other research organisms

Policy information about studies involving animals; ARRIVE guidelines recommended for reporting animal research, and Sex and Gender in Research

Laboratory animals	Sprague Dawley rats, 12-week olds
Wild animals	The study did not involve wild animals
Reporting on sex	Female rats were used for this study due to their docile behaviors and no study has shown sex-dependent nerve regeneration.
Field-collected samples	The study did not involve samples collected from the field.
Ethics oversight	All animal experiments were approved by UCR IACUC (20180021) prior to experiments.

Note that full information on the approval of the study protocol must also be provided in the manuscript.
